# Understanding HPV-Induced Cancers and Investigating the Barriers Faced by Low- and Middle-Income Countries in Prevention and Treatment

**DOI:** 10.3390/ijms26125581

**Published:** 2025-06-11

**Authors:** Zahab N. Aleezada, Ishika Patel, Nabiha Yusuf

**Affiliations:** Heersink School of Medicine, University of Alabama at Birmingham, Birmingham, AL 35294, USA

**Keywords:** HPV, cervical cancer, LMICs, vaccine access, screening, senolytics, public health, healthcare disparity, prevention, global oncology

## Abstract

Human papillomavirus (HPV) is a leading cause of several cancers, most notably cervical cancer, but also anal, penile, vulvar, vaginal, and oropharyngeal malignancies. While vaccines and screening technologies offer highly effective prevention, the global burden of HPV-induced cancers remains disproportionately high in low- and middle-income countries (LMICs). This literature review provides a comprehensive synthesis of the types, mechanisms, treatments, and prevention strategies associated with HPV-related cancers, while also highlighting regional disparities in healthcare access and infrastructure. It critically examines the barriers LMICs face in adopting life-saving interventions, such as limited healthcare infrastructure, vaccine hesitancy, funding gaps, and cultural stigma. The review further explores recent scientific and policy advances—including single-dose vaccination, self-sampling HPV tests, and senolytic therapies—that have the potential to reduce global health inequities. By connecting molecular biology with public health systems, this paper underscores the need for interdisciplinary solutions and equity-centered approaches to combat HPV-induced cancers worldwide. The findings emphasize that eliminating cervical cancer and other HPV-related diseases is not only a scientific goal but also a moral imperative requiring global collaboration and local action.

## 1. Introduction

Human papillomavirus (HPV) is a well-established etiological factor for multiple types of cancers, most notably cervical cancer. However, increasing evidence highlights its role in a range of anogenital and oropharyngeal malignancies [1]. HPV and its various subtypes come from the viral family *Papillomaviridae*. These viruses are non-enveloped, and their genome structure consists of double-stranded DNA, which is then encased by an icosahedral capsid. Due to the structural nature of the virus, it is highly tissue-specific and infects both cutaneous and mucosal epithelia [2]. Among the various known HPV types, a subset of high-risk types—particularly HPV-16 and HPV-18—are responsible for the majority of cervical cancer cases worldwide [3]. The classification of HPV-induced cancers is crucial for understanding their distinct pathological features, guiding prevention efforts, and tailoring treatment strategies [4].

## 2. Epidemiology

HPV is one of the most common sexually transmitted infections worldwide, with an estimated 85% of sexually active women and 91% of men acquiring the virus during their lifetime [5]. Globally, HPV is responsible for approximately 5% of all cancers, including virtually all cases of cervical cancer and a significant proportion of anal, penile, vulvar, vaginal, and oropharyngeal cancers [6]. The World Health Organization reports that cervical cancer alone accounts for approximately 342,000 deaths annually, the vast majority of which occur in low- and middle-income countries (LMICs) [7].

The prevalence and impact of HPV-related cancers vary significantly by region and gender. In sub-Saharan Africa, cervical cancer is the leading cause of cancer-related mortality among women due to limited access to screening and vaccination [8]. Meanwhile, in high-income countries, oropharyngeal cancers—particularly among men—are increasing and now surpass cervical cancer as the most common HPV-associated cancer in some populations [5]. These trends reflect both differences in sexual health behavior and gaps in access to preventive services.

Meta-analyses have shown that the prevalence of HPV among women with normal cytology can range from 1.6% to 42%, depending on the geographic location, with the highest rates reported in Africa and Latin America [9]. Among men, the global prevalence of genital HPV infection is also high, with regional variation and underreporting due to limited male-focused screening initiatives [10]. Certain high-risk populations—such as men who have sex with men (MSM), people living with HIV, and sex workers—demonstrate elevated HPV infection rates and are at an increased risk for cancer progression [11]. 

Disparities in healthcare infrastructure and surveillance systems often result in the underreporting and misclassification of HPV-associated cancers in LMICs. Many countries lack national cancer registries or routine cervical cancer screening programs, making it difficult to fully capture the disease burden [12]. Additionally, social, cultural, and educational barriers can deter women from seeking screening, further exacerbating disease progression and mortality. The absence of data not only masks the true extent of the crisis but also limits resource allocation and policy development [13,14]. 

In sum, the epidemiology of HPV-induced cancers underscores stark global health inequities. While high-income countries are advancing toward the elimination of cervical cancer, LMICs bear a disproportionate share of the morbidity and mortality [5,8]. Understanding these disparities is crucial for designing targeted interventions. The next section will examine the socioeconomic, cultural, and structural barriers that hinder HPV prevention and treatment efforts in low- and middle-income countries.

## 3. Multilevel Barriers to HPV Prevention and Treatment in LMICs

Despite the availability of effective vaccines, screening methods, and treatment strategies, LMICs continue to bear the greatest burden of HPV-related cancers. A significant number of cervical cancer deaths occur in these regions [15]. This disparity stems from multiple systemic factors: weak healthcare infrastructure, underfunded immunization programs, shortages of trained personnel, and a lack of integrated cancer surveillance systems. These limitations prevent the timely detection and management of HPV-induced disease, contributing to late-stage diagnoses and high mortality rates [16].

Financial barriers are among the most pervasive issues. Many LMICs struggle with the costs associated with cold chain storage, transportation, and ensuring a consistent vaccine supply, especially in rural areas [8,16,17]. HPV vaccines—though often subsidized through initiatives like Gavi—still face funding gaps when national health budgets are strained [18]. Inconsistent funding and poor procurement planning often lead to vaccine shortages or delayed rollouts. Screening tools like HPV DNA tests are also expensive, limiting their availability to urban centers [16].

Deeply rooted cultural stigmas and gender inequities also impede prevention and care efforts. In many LMICs, HPV and cervical cancer are poorly understood, and discussions about sexual health are often taboo [9]. Misinformation surrounding HPV vaccines—such as fears about fertility or promiscuity—has led to vaccine hesitancy among parents and adolescents. Furthermore, many women lack the autonomy to make health decisions independently, reducing the uptake of both vaccines and screening services [19].

**Figure 1:**
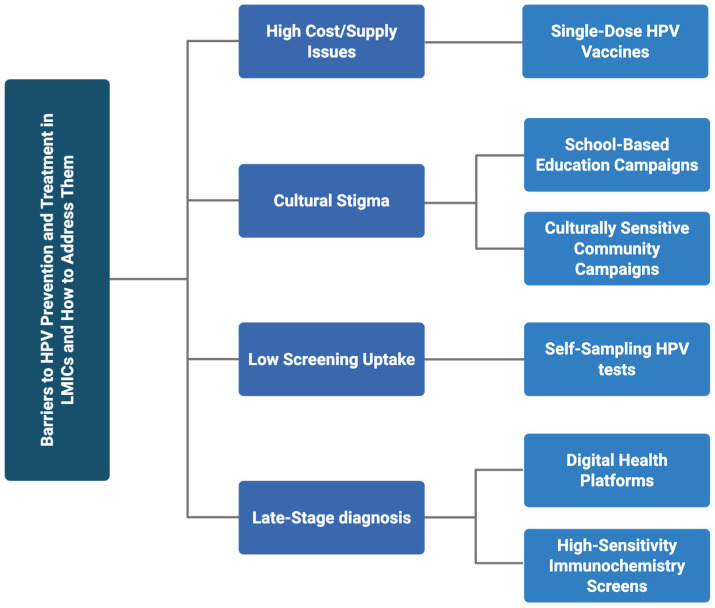
Barriers and solutions to HPV prevention and treatment in LMICs.

A lack of reliable data hampers effective intervention. Many LMICs do not have functioning cancer registries or routine monitoring systems, which makes it difficult to measure the vaccine effectiveness, track the incidence, or identify at-risk groups [16]. This contributes to underreporting and the invisibility of HPV-related diseases in national policy discussions. Additionally, health systems often prioritize acute infectious diseases like HIV and tuberculosis, leaving chronic preventable diseases like cervical cancer under-resourced [20]. These barriers and potential improvement initiatives are summarized in Figure 1.

While these barriers reflect structural and cultural challenges common across LMICs, it is also critical to explore how they play out in specific national contexts. The following examples from Mexico, Nigeria, and Saudi Arabia illustrate the varied but interlinked obstacles to effective HPV prevention and care.

A study conducted in Mexico demonstrated that a significant shift is occurring in the screening methods used for cervical cancer. While Pap smears have historically been the primary tool for early detection, their limitations have prompted clinicians to explore more advanced and reliable methods. A striking finding was that 22.1% of Pap-negative results were actually HPV-positive, revealing a considerable overcall rate and indicating missed opportunities for early intervention. As a result, healthcare providers are now prioritizing newer techniques such as Pap cytology, high-risk HPV DNA PCR, and p16/Ki-67 dual immunocytochemistry, which have demonstrated higher sensitivity, improved accuracy, and cultural acceptability. These technologies also present a more cost-effective approach in the long term, reducing the burden of repeated or inaccurate screenings. However, despite their promise, barriers such as uneven regional access, a lack of skilled personnel, and limited funding remain critical challenges to scaling these tools across all Mexican states [21].

In Nigeria, access to HPV vaccination is still limited, with no national provision of free vaccines. A cross-sectional study conducted among students and staff at Ibrahim Badamasi Babangida University revealed a high incidence of HPV infections, especially among women aged 26–40. The study also highlighted that a significant portion of participants lacked awareness of HPV and its related health risks, even within an academic setting. Furthermore, abject poverty, a lack of organized medical services, and the absence of national policies mandating HPV vaccination have all contributed to the widespread prevalence of HPV-related conditions. Without comprehensive public education or subsidized healthcare infrastructure, preventive efforts remain underutilized. The study’s authors advocate for HPV awareness programs and policy reforms to introduce free HPV vaccines in Nigeria, emphasizing that public knowledge must be improved in tandem with vaccine accessibility [22].

In Saudi Arabia, despite the availability of the HPV vaccine since 2017 and its incorporation into the national immunization program in select regions, the uptake remains alarmingly low at just 7.6% [23]. A recent mapping review attributed this to pervasive cultural stigma, limited sexual and reproductive health education, and misconceptions about the vaccine’s safety and necessity [24]. Prior to 2022, topics such as HPV and cervical cancer were absent from school curricula, and even today, awareness among both the general public and healthcare providers remains low. Studies have indicated that 84.1% of women surveyed had no knowledge of cervical cancer screening, and vaccine hesitancy extended even to medical students [23,25]. While HPV vaccines have been approved, a lack of routine screening programs and insufficient educational campaigns exacerbate the issue. Community attitudes—shaped by religion, tradition, and misinformation—must be addressed through culturally sensitive public health interventions if the vaccine uptake is to improve and the cervical cancer incidence is to decline [26].

In summary, LMICs face a complex web of challenges in addressing HPV-related cancers, from infrastructure and affordability to misinformation and social stigma. By examining both cross-cutting issues and localized case studies, it becomes clear that solutions must be multidimensional and context-specific. Addressing these barriers requires multi-sectoral collaboration, sustained political will, culturally competent public health education, and community engagement.

## 4. Types of HPV-Induced Cancers

HPV is most commonly associated with cervical cancer, which remains the fourth most common cancer among women globally [15]. While cervical cancer is often discussed due to its screening protocols and vaccine relevance, non-cervical HPV-induced malignancies are increasingly recognized in both males and females. Other well-documented HPV-related cancers include vaginal, vulvar, penile, and anal cancers [1]. Despite their rarity, the incidence of vaginal and vulvar cancers has increased. Furthermore, 78% of vaginal and 25% of vulvar malignancies have been associated with HPV [6]. Penile and anal cancers have similarly been liked with HPV; 45.5% of penile and 90% of anal malignancies tested positive for HPV DNA [27]. More recently, HPV has also been implicated in a growing number of oropharyngeal squamous cell carcinomas, particularly in younger, non-smoking populations [28].

The burden of HPV-induced cancers varies by region and gender. Cervical cancer disproportionately affects women in low- and middle-income countries (LMICs), where screening and vaccination programs are often limited [29]. Conversely, the rise in HPV-related vaginal, vulvar, anal, and oropharyngeal cancers is more prominent in high-income countries [6,28,30]. While anal cancer is more prominent in women, oropharyngeal cancer associated with HPV is more evident in males [28,31]. These epidemiological patterns underline the importance of both gender-neutral vaccination strategies and tailored regional interventions.

In summary, HPV is implicated in a diverse group of cancers that affect multiple anatomical sites and span across genders. While cervical cancer remains the most studied, emerging trends in anogenital cancers signal the expanding impact of HPV. Understanding the types of cancers linked to HPV sets the foundation for exploring the biological mechanisms of oncogenesis, which are discussed in the next section.

## 5. Mechanism of HPV-Induced Cancers

Human papillomavirus (HPV)-induced carcinogenesis is driven by persistent infection with high-risk HPV types, most notably HPV-16 and HPV-18. While most infections clear spontaneously, in some cases the dysregulation of E6 and E7 expression leads to malignant transformation, which can be achieved if the virus maintains episomal HPV DNA, integrates into host DNA, or both [32,33]. Understanding the molecular mechanisms through which HPV contributes to cancer development is critical to informing treatment strategies and prevention initiatives, especially in LMICs where HPV-related cancers remain a significant burden [15,34].

At the center of HPV-mediated oncogenesis are the viral oncoproteins E6 and E7, which inactivate tumor suppressor genes. E6 binds to and degrades the p53 protein, impairing DNA damage repair and apoptosis, while E7 inactivates the retinoblastoma protein (pRb), leading to unchecked cellular proliferation [32,35]. These disruptions interfere with normal cell cycle checkpoints, enabling genomic instability and cellular transformation. The persistent expression of E6/E7 is necessary for tumor maintenance, making them critical therapeutic targets [35]. Of note, the mechanisms highlighted here represent only a subset of the many processes by which HPV interferes with the host’s immune responses. Comprehensive reviews of this specific topic explore this topic in greater detail [36,37,38].

HPV also facilitates immune evasion by downregulating key components of the host’s innate and adaptive immune systems, contributing to persistent infection. Senescence—a stable form of growth arrest—is another mechanism implicated in HPV-induced carcinogenesis. While it serves as a protective mechanism against tumor formation, HPV can exploit senescence pathways to evade apoptosis. The senescence-associated secretory phenotype (SASP) creates a pro-inflammatory environment that may paradoxically promote tumor progression [39,40].

Beyond viral factors, epigenetic modifications and host co-factors such as immunosuppression, hormonal influences, and co-infection with other sexually transmitted infections play significant roles in modulating the risk of progression [41,42]. LMIC populations often face increased exposure to these co-factors due to barriers in healthcare infrastructure and access to preventive care, thus intensifying the effects of HPV-mediated transformation [7,16].

In summary, the carcinogenic mechanism of HPV hinges on its ability to hijack host cellular machinery, particularly through the E6 and E7 oncoproteins. These molecular events, along with immune modulation and host co-factors, underpin the development of cervical and other HPV-related cancers [32,35]. This foundational understanding paves the way for exploring treatment options and innovations, which will be addressed in the following section.

## 6. Treatment

The treatment of HPV-induced cancers depends largely on the anatomical site, stage at diagnosis, and available healthcare infrastructure. Cervical cancer, the most common HPV-related malignancy, is typically treated using a combination of surgery, radiation therapy, and chemotherapy [43,44]. For early-stage disease, surgical excision (e.g., conization or hysterectomy) is often curative. In more advanced cases, concurrent chemoradiation is the standard of care. The treatment of non-cervical HPV-associated cancers, such as anal or oropharyngeal cancers, follows similar protocols tailored to their respective anatomical and clinical characteristics [9].

In recent years, there has been growing interest in immunotherapy as a treatment option for HPV-related cancers. Therapeutic vaccines and immune checkpoint inhibitors, particularly PD-1/PD-L1 blockers, have shown promise in clinical trials [45,46]. These strategies aim to harness the immune system to recognize and destroy HPV-infected cells, which typically express viral antigens like E6 and E7 [46]. While still emerging, these approaches offer hope for improving outcomes, particularly in recurrent or metastatic disease.

A significant challenge in HPV cancer management is the development of resistance to conventional therapies. This is often driven by cellular senescence and immune escape mechanisms [47]. Tumor cells can enter a senescent-like state in response to treatment, avoiding apoptosis while remaining metabolically active. Recent research suggests that targeting senescent cells using senolytic agents may enhance the treatment efficacy and overcome resistance [48]. This represents a novel and promising direction for managing advanced cervical cancer.

Despite these advancements, disparities in treatment access persist globally, particularly in LMICs. Limited access to diagnostic services, trained oncologists, radiation facilities, and consistent medication supply chains hampers optimal care delivery [16]. In many settings, women are diagnosed at later stages, leading to poorer outcomes and reduced chances of curative treatment [49]. These systemic gaps underscore the need for investments in healthcare infrastructure, task-shifting strategies, and affordable therapies.

In summary, treatment for HPV-induced cancers encompasses traditional modalities such as surgery and chemoradiation, as well as emerging approaches like immunotherapy and the use of senolytic agents. Addressing therapeutic resistance and improving equitable access to care, especially in LMICs, are key priorities. The next section will examine prevention strategies, which remain the most cost-effective and transformative tools in reducing the HPV-related cancer burden.

## 7. Methods of Prevention

The prevention of HPV-induced cancers relies on two foundational strategies: prophylactic vaccination and early detection through screening. These interventions are highly effective and cost-efficient, especially when implemented systematically [50]. Vaccination can prevent up to 90% of cervical cancers, while routine screening allows for the detection and treatment of precancerous lesions before they progress to malignancy [9]. Together, these tools form the cornerstone of global efforts to reduce the burden of HPV-related disease.

Screening for HPV-induced cancers has evolved significantly in recent years. The traditional Papanicolaou (Pap) smear remains widely used, particularly in high-income countries, to detect cytological abnormalities. However, HPV DNA testing has emerged as a more sensitive alternative, capable of detecting high-risk HPV types before cytological changes appear [9,51]. Visual inspection with acetic acid (VIA) is commonly used in LMICs due to its low cost and simplicity, although it has lower sensitivity [52]. Molecular HPV testing is increasingly being incorporated into national screening programs, sometimes in self-sampling formats to increase the coverage and reduce stigma [53,54].

Vaccination against HPV is the most effective long-term strategy to prevent HPV-related cancers. Vaccines like Cervarix, Gardasil, and Gardasil 9 offer protection against high-risk types, especially HPV-16 and HPV-18, which are implicated in the majority of cases [55,56,57]. Single-dose regimens are now being explored to increase their feasibility in LMICs [58]. However, vaccine rollout faces challenges including supply limitations, vaccine hesitancy, infrastructure deficits, and difficulties in reaching out-of-school adolescents [8].

Disparities in access to both vaccines and screening remain a significant barrier to progress in LMICs. Many countries still lack national HPV vaccination programs or have limited coverage due to costs, logistics, or misinformation. Additionally, women in rural or marginalized communities may lack access to clinics or education about the importance of screening [5,8,12]. Innovative strategies such as mobile clinics, community health workers, and school-based programs are increasingly being adopted to address these inequities [12,59,60].

Overall, preventing HPV-induced cancers hinges on effective vaccination and widespread screening using reliable testing methods. While high-income countries are making progress toward HPV-related cancer elimination, LMICs continue to face significant structural and resource-related challenges. These realities highlight the urgency of strengthening prevention frameworks and health equity. The next section will explore recent advances that aim to close these gaps and enhance prevention, treatment, and access on a global scale.

## 8. Recent Advances

One of the most significant recent advances is the validation of single-dose HPV vaccines, which offer protection comparable to that provided by multi-dose regimens. This change, endorsed by the WHO, holds major promise for expanding the vaccine coverage in LMICs where logistical and financial challenges have previously hindered the uptake [58]. Additionally, newer vaccines like Gardasil 9 extend protection to five additional high-risk HPV types beyond HPV-16 and HPV-18, which are responsible for the majority of cervical cancer cases [56,57].

Screening technologies have also seen a shift toward high-sensitivity, low-cost innovations. Self-sampling for HPV DNA testing is increasingly being adopted, especially in culturally conservative or rural communities. These methods show high levels of accuracy and patient acceptability, particularly when paired with community outreach or mobile health initiatives [54]. Moreover, digital health platforms are being piloted to improve tracking, ensure follow-up care, and deliver population-level insights in real time, enhancing the infrastructure for cervical cancer surveillance and intervention [61].

On the therapeutic front, emerging senolytic therapies are under investigation for their ability to eliminate therapy-resistant senescent cells in HPV-positive tumors, showing promise in mitigating relapses and improving the long-term outcomes [48]. Immunotherapies—including checkpoint inhibitors and therapeutic HPV vaccines—are also advancing. These treatments are showing favorable outcomes in clinical trials targeting persistent or metastatic HPV-positive cancers [45,46].

A recent study has shed light on the role of the HPV16 E6 oncogene in immune evasion. Researchers have identified a novel PD-L1/miR-143/HIF-1α pathway by which E6 facilitates immune suppression in cervical cancer. Through this mechanism, E6 reduces the expression of miR-143, a tumor-suppressing microRNA that normally inhibits HIF-1α, a transcription factor known to upregulate PD-L1, an immune checkpoint inhibitor. The upregulation of PD-L1 in tumor cells impairs T-cell activation, allowing the cancer to evade immunosurveillance. When E6 was knocked out in cervical cancer cell lines, researchers observed decreased PD-L1 and HIF-1α expression and restored levels of miR-143, strongly suggesting that E6 is central to cervical cancer’s immune escape phenotype [34]. This discovery opens up new potential for targeted therapies, such as PD-L1 inhibitors and miRNA-based treatments.

At the policy level, the WHO’s Cervical Cancer Elimination Initiative is increasing the global momentum, aiming to reduce the incidence to fewer than 4 cases per 100,000 women [62]. Meanwhile, Gavi-backed pooled procurement programs and regional vaccine manufacturing hubs are improving vaccine affordability and accessibility [18]. School-based immunization campaigns, combined with culturally sensitive education efforts, are also helping to address vaccine hesitancy and misinformation [8,12].

These recent advances—from single-dose vaccines and digital diagnostic tools to molecular immunology breakthroughs and expanded epidemiological insights—represent a major turning point in HPV research and intervention. The discovery of immune evasion mechanisms facilitated by HPV16 E6 underscore the need to continually evolve our strategies. Bridging these scientific insights with practical implementation will be critical to achieving equity in prevention, diagnosis, and treatment across all regions of the world.

## 9. Gaps in Research on HPV-Induced Cancers

**Figure 2:**
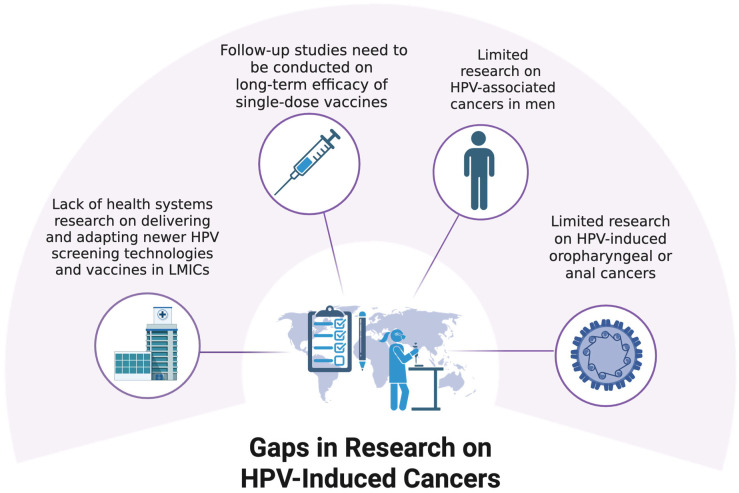
Gaps in research on HPV-induced cancers.

Despite remarkable progress in HPV research, several key gaps remain, particularly in how findings are translated into equitable global health outcomes. Most clinical trials for HPV vaccines and therapeutics are conducted in high-income countries, leaving uncertainties about their real-world effectiveness in LMICs [5,8]. Additionally, the long-term efficacy of single-dose vaccines, although promising, requires further validation through population-based follow-up studies [58]. There is also limited research on HPV-associated cancers in men and in areas of the body outside the female reproductive tract, such as oropharyngeal or anal cancers in underrepresented communities [10]. These gaps are summarized in Figure 2.

In the literature, the implementation barriers in LMICs continue to be under-explored. While HPV vaccines and screening tools are available, little has been published on how to adapt these technologies to local realities—logistics, misinformation issues, workforce limitations, and sociocultural stigma. Health systems research that bridges technical efficacy and practical delivery in low-resource contexts is still scarce, and most global cancer strategies remain inadequately informed by LMIC-specific data [8,63,64].

This review directly addresses some of these critical gaps by synthesizing the literature not just on the biomedical mechanisms of HPV-induced carcinogenesis but also on the systemic inequalities in prevention and treatment delivery. It uniquely connects molecular science to health system barriers, highlighting how innovations such as senolytics, single-dose vaccines, and self-sampling testing methods can transform outcomes if structural inequities are addressed. By focusing on LMICs, this paper brings to light underserved populations often missing from the center of HPV discourse.

The global burden of HPV-induced cancers is not just a virological or oncological issue—it is a justice issue. Addressing the disproportionate effect HPV-induced cancers have in LMICs requires more than just biomedical solutions: it requires interdisciplinary research, inclusive policies, sustained funding, and culturally tailored public health campaigns. Highlighting these intersecting themes helps reposition cervical and other HPV-related cancers as both preventable diseases and indicators of broader health system failures.

In conclusion, this literature review bridges the molecular, epidemiological, and sociopolitical dimensions of HPV-induced cancers. By identifying existing gaps and highlighting scalable innovations, it reinforces the urgency for global efforts to eliminate HPV-related cancers, not only through vaccines and technologies but also by dismantling structural barriers in LMICs. The road to elimination is not purely scientific—it is moral, and it must be equitable.
